# Integer programming for improving radiotherapy treatment efficiency

**DOI:** 10.1371/journal.pone.0180564

**Published:** 2017-07-10

**Authors:** Ming Lv, Yi Li, Bo Kou, Zhili Zhou

**Affiliations:** 1 School of Management, Xi’an Jiao Tong University, Xi’an, Shaan Xi, P.R. China; 2 Department of Radiation Oncology, The First Affiliated Hospital, Xi’an Jiao Tong University, Xi’an, Shaan Xi, P.R. China; 3 Department of Cardiac Surgery, The First Affiliated Hospital, Xi’an Jiao Tong University, Xi’an, Shaan Xi, P.R. China; University of California Davis, UNITED STATES

## Abstract

**Background and purpose:**

Patients received by radiotherapy departments are diverse and may be diagnosed with different cancers. Therefore, they need different radiotherapy treatment plans and thus have different needs for medical resources. This research aims to explore the best method of scheduling the admission of patients receiving radiotherapy so as to reduce patient loss and maximize the usage efficiency of service resources.

**Materials and methods:**

A mix integer programming (MIP) model integrated with special features of radiotherapy is constructed. The data used here is based on the historical data collected and we propose an exact method to solve the MIP model.

**Results:**

Compared with the traditional First Come First Served (FCFS) method, the new method has boosted patient admission as well as the usage of linear accelerators (LINAC) and beds.

**Conclusions:**

The integer programming model can be used to describe the complex problem of scheduling radio-receiving patients, to identify the bottleneck resources that hinder patient admission, and to obtain the optimal LINAC-bed radio under the current data conditions. Different management strategies can be implemented by adjusting the settings of the MIP model. The computational results can serve as a reference for the policy-makers in decision making.

## Introduction

Normally, there is a period between a patient’s first-time consultation and the beginning of the radiotherapy [1]. Research suggests that the waiting time is relatively long [2–4]. The admission of patients receiving radiotherapy is an admission scheduling problem (ASP). It means that the patients are notified of whether and when they can be admitted by the oncology centers several days before their admission [5]. In China, because oncology centers concentrate in middle-size and large-size cities, famous oncology centers are usually overcrowded with patients. Naturally, some patients may not be able to be admitted within a certain period of time. In that case, they can either choose to wait or to go to another oncology center. Whatever choice they make, the treatment is delayed more or less (Some studies show that there is a correlation between the length of waiting time and outcomes of treatment for radio-receiving patients [6–8]). Here is the question for the oncology centers: What should be done to admit more patients in a specific period?

There are two lines of thinking for solving this problem. One comes from the medical world, including improvement of the existing treatment technologies [9–11] and the introduction of new treatment facilities [12, 13]. The other is formed from the management science perspective, including the introduction of statistics [14] and operations research [15–24] to boost the work efficiency of personnel and facilities.

**Fig 1:**
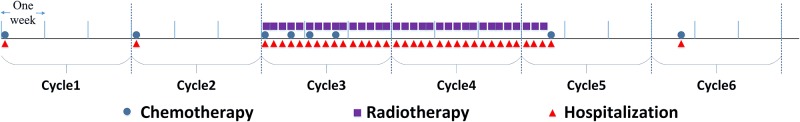
Treatment pathway of a radio-receiving patient who have nasopharynx cancer.

In practice, it seems unrealistic to improve the existing treatment technologies and equipment within a short period of time. Therefore, the latter option seems to be a more practical choice. In management academia, operations research is the most commonly used method of solving such problems [25]. It has been widely applied to scheduling and queuing problems in manufacturing, communications, and traffic. Some scholars have used operational research to solve the ASP problem [26–28]. However, the problem of radio-receiving patient admission is different from ordinary outpatient clinic or emergency treatment, because each radio-receiving patient will be assigned with a treatment plan, which is formulated by the physician team according to the type, location, size of the tumor as well as the patient’s health conditions. The treatment plan stipulates in detail the dates and form of radiotherapy given to the patient. In other words, radio-receiving patients would be admitted regularly for several times (An example of PFL treatment plan of nasopharynx cancer can be seen in Fig 1). Different treatment plans involve different treatment time length and modes, which could encompass different demands for medical service resources. Some treatment plans include only radiotherapy while some include both radiotherapy and chemotherapy, which makes the situation more complicated. Some patients do not need to stay in hospital but are required to be hospitalized (depending on the requirements of the treatment plan, patient’s health conditions and medical insurance policies, etc.). In China, most RT patients are in-patients. However, in the U.S., most RT patients are out-patients. Therefore, the admission of radio-receiving is more complex than the traditional ASP problem. It is difficult to copy solutions from the traditional ASP problem or similar problems (like multi-resource assignment problem and the resource constrained project scheduling problem with minimal and maximal time-lags). We need to find new solutions to solve this problem.

## Materials and methods

We made statistics of patients admitted by the oncology center of the First Affiliated Hospital of Xi’an JiaoTong University from August 31th to September 27th, 2015 (four consecutive weeks) by retrieving the admission record, and sending personnel to oncology center for data collection. As part of the data is missing and does not meet the assumptions, we re-sort the data, based on which we construct calculation examples needed in computational experiments.

We classify patients into two types, namely booked patients and waiting patients. Booked patients include the patients who have started treatment and some emergency patients. Their needs must be met as priority. Waiting patients are those who have not started treatment yet. They may be admitted or denied admission. We list nine cancer treatment plans (see Table 1), each sub-plan of the nine treatment plans can be seen as a new treatment plan. The treatment plans for certain cancers are differently. But they encompass the same usage of LINAC and bed occupancy. Therefore, these nine basic treatment plans are able to cover most cases of patients’ conditions. Similarly, we consider only three basic radiotherapy treatment means, namely, Conformal Radiation Therapy(CRT), Intensity Modulated Radiation Therapy(IMRT) and 2-Dimension Radiation Therapy (2DRT), which also cover most cases of patients’ treatment.

**Table 1:** Treatment plans used in this article.

No	cancer name	Protocol(chemotherapy+radiation)
**1**	nasopharynx cancer	PFL(Cisplatin+5-Fluororacil+leucovorin)+7029cGy/33fraction
**2**	laryngeal cancer	TP(Docetaxel+Cisplatin)+50000cGy/25fraction
**3**	lung cancer	GP(Gemcitabine+Sisplatin)+6000cGy/30fraction
**4**	breast cancer	ACT(Doxorubicin+Cyclophosphamide+Paclitaxel)+5000cGy/25fraction
**5**	gastric cancer	TPF(Paclitaxel+Cisplatin+5-Fluorouracil)+5000cGy/25fraction
**6**	cervical cancer	TP(Paclitaxel+Cisplatin)/PF(Cisplatin+5-Fluorouracil)+5000cGy/25fraction
**7**	prostate cancer	No+7000cGy/35fraction
**8**	liver cancer	No+3000cGy/15fraction
**9**	basal cell carcinoma	No+6000cGy/30fraction

Integer programming problem is a linear and non-linear programming problem which requires the decision variables to take integer values. It is an important branch of operational research and optimization theory. The model, theory and algorithm of integer programming are widely applied in the fields of management science, economics, financial engineering, IT industry management and so on. In the previous studies of appointment scheduling problem, the integer programming method is used by the majority of experts to build the model. Then, we construct an integer programming model for solving the ASP problem at the oncology center (see S1 File). Normally, when constructing the mathematical model, we need to make certain assumptions to simplify the problem. As to this problem, we made the following assumptions.

Firstly, we assume that the decisions on patient admission are made at an interval instead of in real time. Therefore, the time window between two decisions concerning patient admission is a decision-making period. We define the set of dates in the decision-making period as |T|. After each time of admission, a patient will produce an impact on the service resources (staffs, medical facilities and beds, etc.) during the period of his subsequent treatment according to his treatment plan. Therefore, when making a decision on admission, we need to consider a substantial large planning horizon that can take into account all possible impacts of patient admission on resources. We define the set of all dates in the planning horizon as |H|. Obviously, T is a subset of H. Secondly, we assume that the length of treatment (including the period of the radiotherapy and the time taken for setup) can be estimated by computer simulation (this assumption is the same as that in Conforti’s study [19]) and that the time length of the first treatment is the same as that of all subsequent treatment. Of course, the length of treatment is related to the type of radiation therapy. Finally, because the oncology department remains closed during weekends, we consider only weekdays so that the influence of weekends can be ruled out when constructing the model.

Apart from the assumptions above which aimed at simplifying the problem, we set the basic parameters in the model as follows according to the collected data:

- About nearly 90% of patients need to be hospitalized (the proportion for the fourth week is nearly 95%).
- There are two LINACs available, namely, M1 and M2. There are two treatment shifts, including one in the morning and one in the afternoon. Each shift lasts for five hours.
- The scheduling period |T| = 5 ((5 work days in a week) while |H| = 5*3*6 (the longest among all treatment plans+|T|).
- All beds are placed in wards. There may be multiple beds in one ward. Patients are identified by their ward number instead of bed number. For the ease of modeling, we assume that if there are |J| wards, then the ward for patients who are not hospitalized are defined as a virtual ward |J|+1.

We set a total of four groups of calculation examples, with each corresponding to a week in September of 2015. The parameter settings of the four calculation examples are shown in Table 2.

**Table 2:** Parameter settings of calculation examples.

Problem instance	Daily available beds	Number of booked patients	Number of waiting patients
**Week1_1**	72	122	70
**Week2_1**	80	124	74
**Week3_1**	88	136	90
**Week4_1**	76	115	56

Considering our purpose to examine the principles underlying the process, real conditions in oncology centers and complexity of the problem, we use the commercial software IBM ILOG CPLEX12.5 to solve the problem. IBM ILOG CPLEX is the most commonly used high-performance mathematical programming solver for linear programming, mixed integer programming, and quadratic programming. This software is able to obtain the exact solution to the problem. The result achieved in this manner is the first calculation example of each group. Besides, in order to compare our method against the existing method, we use computer program to simulate the currently used method of scheduling patients. Normally, oncology center uses the FCFS method to schedule patient admission, a method similar to the greedy algorithm. Specifically, it means patients are admitted only when there are available beds. Otherwise, patients can choose to either wait or to go to another oncology center. We use MATLAB to do the simulation. It is run 1000 times for each calculation example. The optimal value is selected as the second calculation example of each group.

## Results

Computational experiments are carried out on a server with Intel Xeon E5 (3.5 GHz, 12 threads) processor and 16GB RAM running Windows 7 Professional operating system. The solver is IBM ILOG 12.5. Computational results are reported in Tables 3 and 4.

**Table 3:** Computation results of standard calculation examples.

Instance	Admission rate	Machine occupancy (in the next week)	Bed occupancy (in the next week)	Machine occupancy (in the next quarter)	Bed Occupancy(in the next quarter)	Computational time (seconds)
**Week1_1**	88.57%	82.87%	94.54%	65.75%	91.90%	117
**Week1_2**	37.14%	76.43%	82.52%	53.65%	72.27%	88
**Week2_1**	64.86%	80.63%	85.50%	64.26%	72.71%	15
**Week2_2**	54.05%	78.42%	82.06%	61.33%	67.17%	89
**Week3_1**	60.0%	75.46%	80.30%	67.46%	73.83%	47
**Week3_2**	44.44%	72.26%	76.10%	57.40%	65.63%	87
**Week4_1**	82.14%	75.19%	80.23%	67.67%	77.22%	72
**Week4_2**	46.43%	74.90%	74.45%	66.85%	64.20%	92

**Table 4:** Minimum remaining service resources in the next quarter.

Instance	Number of beds	M1 morning (min)	M1 afternoon (min)	M2 morning (min)	M2 afternoon (min)
**Week1_1**	5	12	3	0	4
**Week2_1**	8	0	1	3	9
**Week3_1**	5	0	1	0	8
**Week4_1**	0	1	2	3	11

According to the results from Table 3, there is always insufficient available time in the first three weeks (equals 0 or 1, less than 3 minutes which is the minimum time required for radiotherapy for the patient). In week four, there is insufficient number of beds. Therefore, we assume that the bottleneck for patient admission for the first three weeks is LINAC and the beds’ number for the week four. But through limited observation of computational results, we cannot determine which kind of resource constrains patient admission. In order to examine the impact of different parameters and management strategies on results, we design some new calculation examples. For the purpose of studying the impact of LINAC work time on patient admission, we set LINAC work time+2 hours, with the third calculation example of each group corresponding to it. For the purpose of studying the impact of the number of beds in the oncology center on patient admission, we set the number of daily available beds+16, with the fourth calculation example of each group corresponding to it. For the purpose of studying the impact of the shift’s constraints on patient admission, we assume that patients can receive radiotherapy in either of the two shifts, with the fifth calculation example of each group corresponding to it. For the purpose of studying the impact of the patient’s successive stays in oncology center, we stipulate that the patient’s bed is changeable, with the sixth calculation example of each group corresponding to it. For the purpose of studying the impact of waiting time on patient admission, we stipulate that all waiting patients can delay enrollment one day at most, with the seventh calculation example of each group corresponding to it. The other computational settings of these examples are the same as those of standard calculation examples of each group. The computational results are as Table 5.

**Table 5:** Computational results of newly added calculation examples.

Instance	Admission rate	Machine occupancy (in the next quarter)	Bed Occupancy (in the next quarter)	Computational time (seconds)
**Week1_3**	97.14%	58.74%	97.59%	433
**Week1_4**	88.57%	66.11%	75.49%	82
**Week1_5**	91.43%	67.17%	94.72%	1078
**Week1_6**	88.57%	66.21%	93.75%	98
**Week1_7**	88.57%	65.79%	90.83%	115
**Week2_3**	78.73%	58.94%	74.79%	161
**Week2_4**	64.86%	64.83%	60.38%	28
**Week2_5**	67.57%	64.97%	71.08%	145
**Week2_6**	64.86%	63.70%	72.62%	16
**Week2_7**	64.86%	65.04%	71.58%	12
**Week3_3**	75.67%	63.54%	80.98%	360
**Week3_4**	60.0%	67.45%	62.82%	147
**Week3_5**	64.44%	68.13%	76.25%	358
**Week3_6**	60.0%	67.40%	74.09%	94
**Week3_7**	60%	67.34%	73.98%	146
**Week4_3**	82.14%	61.14%	73.85%	144
**Week4_4**	96.43%	69.89%	64.94%	49
**Week4_5**	82.14%	68.33%	73.67%	73
**Week4_6**	82.14%	68.05%	74.28%	163
**Week4_7**	82.14%	66.87%	73.58%	165

## Discussion

We first compare the MIP method and the traditional FCFS method, that is, the first and the second calculation examples of each group. By comparing the results in Table 2, we find that compared with manual scheduling of patients, the MIP method is able to significantly increase the admission rate and thus increase the LINAC usage and bed occupancy. In all calculation examples, the solutions obtained by using the MIP method are far better than those obtained by manually scheduling patients. Hence, we come to the conclusion that the MIP method is superior over the manual scheduling method. In fact, this is in accordance with expectation. Because the solution obtained by using the MIP method is a global optimum. In contrast, the FCFS method is similar to the greedy algorithm and gives no consideration to global optimization. So, the solution obtained by using the FCFS method cannot be better than that obtained by using the MIP method.

By comparing the third and fourth calculation examples against the first in each group, we can learn about how the changes in LINAC work time and the number of available beds affect results. Results show that as the LINAC work time increases, the admission rate of the third calculation example in the first three groups is higher than their respective standard calculation example while the fourth group remains unchanged. As the number of available beds increases, the admission rate in the first three remains unchanged while the admission rate in the fourth calculation example increases significantly. Therefore, we come to the conclusion that the insufficiency of the LINAC’s available work time is the resource bottleneck for the first three weeks and that the insufficiency of beds is the resource bottleneck for the fourth week. Because we increase the supply of the bottleneck resource only slightly, the number of newly admitted patients does not increase significantly. Consequently, the growth in the usage of the other resource is insignificant.

When the LINAC work time is insufficient, we can consider increasing the number of shifts available to patients apart from directly increasing the work time of LINAC. By comparing the fifth calculation example in the first three calculation examples against the stand example of each group, we find this approach feasible. Computational results show that when both shifts are available to patients, the admission rate can increase slightly. However, as the number of newly admitted patients is small, the service resource usage is only slightly higher than that of the MIP solution of standard example.

When the beds number are insufficient, another line of thinking is to allow patients to change beds during the scheduling period apart from directly increasing the number of beds. We illustrate this condition with the sixth calculation example of each group. Through the observation of the results of the fourth group of calculation examples, we find that the elimination of the constraint of fixed beds has no significant impact on the results. Moreover, while the admission rate remains unchanged, bed occupancy and LINAC’s usage efficiency even may suffer a minor decline. It means that changing this constraint cannot achieve the expected results. Considering the change of this constraint will greatly increase the complexity of the work, it is not worth making this change for hospitals’ senior management.

At last, we discuss a strategy which is to alleviate the problem by delaying patient admission in the face of resource bottlenecks. However, by comparing result of the seventh calculation example of each group and their respective MIP solution of standard example, we find that this approach has no effect at all, because the three measurements remain almost unchanged. This result tells us that there is no increase in resource usage efficiency through prolonging on the basis of original time of the patients. Therefore managers of oncology centers should admit patients as soon as possible, which represents a win-win situation for oncology centers and patients.

## Conclusion

This paper first studies the problem of scheduling patients at the radiotherapy department and constructs a mixed integer programming model for solving this problem. Computational results suggest that compared with the FCFS method, our proposed MIP method is able to significantly increase patient admission and thus boost the usage efficiency of medical service resources (LINAC and bed) by a wide margin.

Furthermore, by adjusting the input parameters in the model and comparing the results after the problem solving, our mathematical model is able to identify the bottleneck resource that hinders patient admission and even obtain the optimal ratio of the two key resources (LINAC and bed) under current circumstances. This will provide a very useful reference for decision making in the management of oncology centers. Moreover, by changing some conditions in the model, we can define different rules for patient admission. Through the comparison and analysis of the solutions, we can learn about the impact of different scheduling rules on patient admission and provide some useful recommendations to aid their decision making process (help their decision making).

## Supporting information

S1 File Appendix. Calculation method.
(DOCX)
