# Supplementary material for: Integer programming for improving radiotherapy treatment efficiency
Source: PLoS One. 2017 Jul 10;12(7):e0180564. doi: 10.1371/journal.pone.0180564 (PMC5503264; doi:10.1371/journal.pone.0180564)
Supplement: S1 File — Calculation method. (DOCX) [file pone.0180564.s001.docx]

# Appendix Calculation method

## Sets

| $P$ | Set of patients that have already begun the treatment course, denoted by $p$ |
| --- | --- |
| $I$ | Waiting list of unscheduled patients ready to start the treatment course, denoted by $i$ |
| $J$ | Set of wards, denoted by $j$ |
| $J^{'}$ | $J^{'}=\{J,\left\vert J \right\vert+1\}$ |
| $H$ | Set of days in the time horizon, denoted by $t$ |
| $T$ | Set of days in the scheduling period, denoted by$t$,$t\in H$ |
| $M$ | Set of linear accelerators with same performance, denoted by $m$ |
| $K$ | Set of treatment plans that need to hospitalized in oncology center, denoted by $k$ |
| $K^{'}$ | Set of treatment plans that do not need to hospitalized in oncology center, denoted by $k'$ |
| $S$ | Set of available shifts according to which each week day is partitioned, denoted by $s$, |
| $WT_{ms}$ | Working time of linear accelerator$m$, during the shift $s$ |
| $HD\{k\}$ | Set of hospitalized days of treatment plan $k$ within a treatment course, denoted by $d$ |
| $RD\{k\}$ | Set of radiation days of protocol $k$ within a treatment course, denoted by $d$ |

## Parameters

| Input parameters: | |
| --- | --- |
|  | |
| $PRT_{p/i}$ | Treatment plan of patient $p/i$ |
| $BSD_{p}$ | Start day of booked patient $p$ |
| $C_{jt}$ | Capacity of ward$j$ on day$t$ |
| $l_{i}$ | Latest start day of patient $i$ |
| $\emptyset_{mp/i}$ | Radiation treatment time of patient $p/i$ on machine $m$ |
| ${SM}_{mp}$ | $=\left\{ \begin{aligned} 1, if booked patient p starts radiation treatment on machine m. \\ 0, otherwise. \end{aligned} \right.$ |
| ${ld}_{i}$ | latest start date of waiting patient $i$ |

| Parameters obtained from input parameters: | |
| --- | --- |
|  | |
| $BQH_{p}$ | Total hospitalized days of booked patient $p$ |
| $WQH_{i}$ | Total hospitalized days of waiting patient $i$ |
| $BQR_{p}$ | Total radiation days of booked patient $p$ |
| $WQR_{i}$ | Total radiation days of waiting patient $i$ |
| $Int(k)$ | The interval time between the first radiation treatment and following radiation treatment in treatment plan $k$ |
| $NAV_{p/i}$ | Set of infeasible shifts of patient $p/i$ |

## Decision variables

| $u_{pst}$ | $=\left\{ \begin{aligned} 1, if patient p recieve treatment duringshift s on day t. \\ 0, otherwise. \end{aligned} \right.$ |
| --- | --- |
| $v_{pjt}$ | $=\left\{ \begin{aligned} 1, if patient p is hopitalized in ward j on day t. \\ 0, otherwise. \end{aligned} \right.$ |
| $x_{mist}$ | $=\left\{ \begin{aligned} 1, if patient i starts treatment on machine m during shift s on day t. \\ 0, otherwise. \end{aligned} \right.$ |
| $y_{mist}$ | $=\left\{ \begin{aligned} 1, if patient i recieves treatment on machine m during shift s on day t. \\ 0, otherwise. \end{aligned} \right.$ |
| $z_{ijt}$ | $=\left\{ \begin{aligned} 1, if patient i is hopitalized in ward j on day t. \\ 0, otherwise. \end{aligned} \right.$ |
| $o_{ijt}$ | $=\left\{ \begin{aligned} 1, if patient i starts treatmetn in ward j on day t. \\ 0, otherwise. \end{aligned} \right.$ |

## Constraints

| $\sum_{s=1}^{\vert S\vert} u_{pst}\leq1$ | $\forall p, \forall t$ | (1) |
| --- | --- | --- |
| $\sum_{s=1}^{\vert S\vert} y_{mist}\leq1$ | $\forall m,\forall i, \forall t$ | (2) |
| $\sum_{s\in NAV_{p}} u_{pst}=0$ | $\forall p, \forall t$ | (3) |
| $\sum_{s\in NAV_{it}} x_{mist}+y_{mist}=0$ | $\forall m,\forall i, \forall t$ | (4) |
| $\sum_{j=1}^{\left\vert J' \right\vert} v_{pjt}\leq1$ | $\forall p, \forall t$ | (5) |
| $v_{p\left( \left\vert J \right\vert+1 \right)t}=0$ | $\forall PRT_{p}\in K, \forall t$ | (6) |
| $\sum_{j=1}^{\left\vert J \right\vert} v_{pjt}=0$ | $\forall PRT_{p}\in K', \forall t$ | (7) |
| $\sum_{j=1}^{\left\vert J' \right\vert} z_{ijt}\leq1$ | $\forall i, \forall t$ | (8) |
| $\sum_{t=1}^{{ld}_{i}} o_{i(\left\vert J \right\vert+1)t}+\sum_{t=1}^{\vert H\vert} z_{i\left( \left\vert J \right\vert+1 \right)t}=0$ | $\forall PRT_{i}\in K, \forall t$ | (9) |
| $\sum_{t=1}^{{ld}_{i}} \sum_{j=1}^{\vert J\vert} o_{ijt}+\sum_{t=1}^{\vert H\vert} \sum_{j=1}^{\vert J\vert} z_{ijt}=0$ | $\forall PRT_{i}\in K', \forall t$ | (10) |
| $\sum_{p=1}^{\vert P\vert} v_{pjt}+\sum_{i=1}^{\vert I\vert} z_{ijt}\leq C_{jt}$ | $\forall j, \forall t$ | (11) |
| $\sum_{p=1}^{\vert P\vert} v_{pjt}+\sum_{i=1}^{\vert I\vert} o_{ijt}\leq C_{jt}$ | $\forall j, \forall t$ | (12) |
| $v_{pjt}\leq v_{pjBSD_{p}}$ | $\forall p, \forall j\in J^{'},\forall t\in\{BSD_{p},\ldots,T\}$ | (13) |
| $Z_{ijt}\leq\sum_{t=1}^{ld_{i}} o_{ijt}$ | $\forall i, \forall j\in J^{'},\forall t\in T$ | (14) |
| $\sum_{s=1}^{\vert S\vert} u_{ps(BSD_{p}+d-1)}=1$ | $\forall p, \forall d\in RD\{PRT_{p}\}$ | (15) |
| $\sum_{j=1}^{\vert J\vert} v_{pj(BSD_{p}+d-1)}=1$ | $\forall PRT_{p}\in K, \forall d\in HD\{PRT_{p}\}$ | (16) |
| $\sum_{m=1}^{\vert M\vert} \sum_{s=1}^{\vert S\vert} \sum_{t=1}^{\vert H\vert} x_{mist}\leq1$ | $\forall i$ | (17) |
| $\sum_{j=1}^{\left\vert J' \right\vert} \sum_{t=1}^{ld_{i}} o_{ijt}\leq1$ | $\forall i$ | (18) |
| $\sum_{j=1}^{\left\vert J' \right\vert} \sum_{t=1}^{\vert T\vert} o_{ijt}\leq1$ | $\forall i$ | (19) |
| $\sum_{t=1}^{\left\vert T \right\vert} \sum_{s=1}^{\vert S\vert} u_{pst}=BQR_{p}$ | $\forall p$ | (20) |
| $\sum_{j=1}^{\left\vert J' \right\vert} \sum_{t=1}^{ld_{i}} o_{ijt}=\sum_{m=1}^{\vert M\vert} \sum_{s=1}^{\vert S\vert} \sum_{t=1}^{\vert H\vert} y_{mist}*WQR_{i}$ | $\forall i$ | (21) |
| $\sum_{t=1}^{\left\vert H \right\vert} \sum_{j=1}^{\vert J\vert} v_{pjt}=BQH_{p}$ | $\forall PRT_{p}\in K$ | (22) |
| $\sum_{j=1}^{\left\vert J \right\vert} \sum_{t=1}^{\vert T\vert} o_{ijt}*WQH_{i}=\sum_{j=1}^{\vert J\vert} \sum_{t=1}^{\vert H\vert} z_{ijt}$ | $\forall PRT_{i}\in K$ | (23) |
| $\sum_{m=1}^{\vert M\vert} y_{mist}\leq1$ | $\forall i,\forall s,\forall t$ | (24) |
| $\sum_{s=1}^{\vert S\vert} y_{mist}\leq\sum_{d=1}^{\vert H\vert} \sum_{s=1}^{\vert S\vert} x_{mist}$ | $\forall m,\forall i,\forall t$ | (25) |
| $\sum_{i=1}^{\vert I\vert} \emptyset_{mi}y_{mist}+\sum_{p=1}^{\vert P\vert} u_{pst}{SM}_{mp}\emptyset_{mp}\leq{WT}_{ms}$ | $\forall m,\forall s,\forall t$ | (26) |
| $\sum_{j=1}^{\left\vert J' \right\vert} o_{ijt}=\sum_{m=1}^{\vert M\vert} \sum_{s=1}^{\vert S\vert} x_{mis(t+Int\left( PRT_{i} \right))}$ | $\forall i,\forall t\in\{1,\ldots,ld_{i}\}$ | (27) |
| $\sum_{j=1}^{\left\vert J' \right\vert} o_{ijt}\leq\sum_{j=1}^{\left\vert J' \right\vert} z_{ij(t+d-1)}$ | $\forall i,\forall t,\forall d\in HD\{PRT_{i}\}$ | (28) |
| $\sum_{j=1}^{\left\vert J' \right\vert} o_{ijt}\leq\sum_{m=1}^{\vert M\vert} \sum_{s=1}^{\vert S\vert} y_{mis(t+d-1)}$ | $\forall i,\forall t,\forall d\in RD\{PRT_{i}\}$ | (29) |
| $\sum_{j=1}^{\left\vert J' \right\vert} \sum_{t=1}^{ld_{i}} o_{ijt}=\sum_{m=1}^{\vert M\vert} \sum_{s=1}^{\vert S\vert} \sum_{t=1}^{\vert H\vert} x_{mist}$ | $\forall i$ | (30) |
| $u_{pst},v_{pjt},x_{mist},y_{mist,}z_{ijt},o_{ijt}\in(0,1)$  ${BSD}_{p},{BQH}_{p},{WQH}_{i},{BQR}_{p},{WQR}_{i},\emptyset_{mp/i},C_{jt},{ld}_{i}\in N$ |  | (31) |

Constraints (1) and (2) specify that patient can be assigned at most one shift every day. Constraints (3) and (4) ensure that patient will not be treated in non-available shifts. Constraints (6)-(12) are bed constraints. Constraints (6) and (9) stipulate that patient who need to hospitalized will not be assigned to virtual ward. Similarly, constraints (7) and (10) guarantee that patient who do not need hospitalized will surely be assigned to virtual ward. To ensure that patient will take at most one ward each day, constraints (8) are formulated. Constraints (11) and (12) mean that the number of patients in a ward will not exceed the ward’s capacity. To ensure that patients will stay in the same ward during the treatment of each session, constraints (13) and (14) are formulated. Constraints (15) and (16) indicate that booked patients’ treatment dates are fixed. Constraints (17)-(19) state that waiting patient can start their treatment at most once. Consequently, taking into account the treatment plans, patients should be treated on specific days, hence the constraints (20)-(23). Constraints (24)-(26) are for LINAC. Constraints (24) specify that patient can be assigned to at most one LINAC each day. Constraints (25) ensure that patient will do radiation treatment on the same LINAC. Constraints (26) mean that the summation of patient treat time will not exceed each LINAC’ working time. Constraints (27)-(30) are for the relationships between decision variables. Constraints (31) are variable constraints.

## Objective function

| $\mathrm{MAX} \sum_{i=1}^{\vert I\vert} \sum_{j=1}^{\left\vert J' \right\vert} \sum_{t=1}^{\vert T\vert} o_{ijt}$ |  | (32) |
| --- | --- | --- |

The goal is to maximize the number of waiting patients who have started treatment. In other words, minimize the patient loss in oncology center.
